# Relationships between irregular pulsation and variations in morphological characteristics during the cardiac cycle in unruptured intracranial aneurysms by 4D-CTA

**DOI:** 10.3389/fneur.2024.1302874

**Published:** 2024-03-28

**Authors:** Shiyao Chen, Nan Lv, Yu Qian, Mingwei Zhang, Tianyi Zhang, Yunzhang Cheng

**Affiliations:** ^1^School of Health Science and Engineering, University of Shanghai for Science and Technology, Shanghai, China; ^2^Cerebrovascular Disease Center, First Affiliated Hospital of Naval Military Medical University, Changhai Hospital of Shanghai, Shanghai, China

**Keywords:** intracranial aneurysms, rupture risk, 4D-CTA, morphology, irregular pulsation

## Abstract

**Background and purpose:**

Irregular pulsation of the aneurysmal wall has been suggested as a novel predictor for aneurysm rupture. Aneurysm volume variations during the cardiac cycle and the association between irregular pulsation and morphological features have been discussed, but the clinical significance remains unclear. The purpose of this study was to quantify changes in morphological characteristics over the cardiac cycle and examine their correlation with irregular pulsation to facilitate comprehension of aneurysm dynamics.

**Materials and methods:**

Fourteen unruptured intracranial aneurysms (UIAs) from 11 patients were included in this study, and each of them underwent 4D-CTA after diagnosis by DSA. The R-R intervals were divided into 20-time phases at 5% intervals to determine whether an aneurysm had irregular pulsation throughout the cardiac cycle. CT images from the 20-time phases were used to reconstruct 3D aneurysm models, measure 14 morphological parameters, and quantify each parameter’s absolute change and relative rates of change during the cardiac cycle.

**Results:**

Seven of 14 UIAs exhibited irregular pulsation over the cardiac cycle by 4D-CTA, 5 of which were small aneurysms ( < 7 mm). The UIAs with irregular pulsation exhibited greater changes in morphological characteristics. As aneurysm size increased, the absolute change in aneurysm volume increased (*p* = 0.035), but the relative rates of change in aneurysm size (*p* = 0.013), height (*p* = 0.014), width (*p* = 0.008), height-to-width ratio (*p* = 0.009), dome-to-neck ratio (*p* = 0.019) and bottleneck factor (*p* = 0.012) decreased.

**Conclusion:**

Although the larger the aneurysm, the greater the amplitude of its volumetric variation, small aneurysms are prone to irregular pulsation during the cardiac cycle and have more pronounced and dramatic morphological changes during the cardiac cycle that may increase the risk of rupture. This proof-of-concept study could help to explain the importance of dynamic changes using 4D-CTA in assessing the rupture risk of UIAs.

## Introduction

Intracranial aneurysm (IA), a prevalent cerebrovascular disease, involves pathological dilation of the cerebral artery typically situated at the branch or bifurcation of the circle of Willis, with an estimated global prevalence of 3.2% (1). Subarachnoid hemorrhage is a severe consequence of aneurysm rupture with high mortality and morbidity rates (2). In clinical practice, determining the optimal management of patients with unruptured IAs (UIAs) remains a point of controversy, mainly due to the exceedingly low annual rupture rate (3) and the non-negligible risk of complications related to prophylactic surgeries (4, 5).

Clinical risk stratifications for aneurysm rupture are primarily based on demographic features (6) and basic morphological features of the aneurysms (7, 8), underestimating the actual rupture risk by ignoring other independent risk factors, such as cigarette smoking, aspect ratio (AR), and size ratio (SR) (9). Furthermore, it is important to note that the shape of an aneurysm is not fixed. Blood flow within the aneurysm undergoes dynamic changes due to the contraction and diastole of the heart. Similar to other cerebral arteries, the aneurysmal wall produces pulsatile motion during the cardiac cycle. Interestingly, for the heterogeneity of the aneurysm wall, the types of aneurysmal wall motion can be global dilation and focal irregular pulsation (10, 11). As a kind of morphology-related cerebrovascular disease, changes in aneurysm morphology may be the result of the interaction between hemodynamics and the aneurysm wall. Therefore, studying aneurysm dynamics during the cardiac cycle may help to further understand aneurysm mechanisms.

Studies on aneurysm dynamics rely on imaging techniques. 4D-CTA combines the non-invasiveness of conventional CTA with the dynamic capability of digital subtraction angiography (DSA) to reliably capture aneurysmal wall motion during the cardiac cycle (12). Although an association between irregular pulsation and aneurysm rupture has been proposed (10, 11, 13), there is still a limited understanding of aneurysm wall motion. Published quantitative research on aneurysm dynamics has been limited to aneurysm volume (14, 15), with a minimal exploration of how other morphological risk factors, such as AR and SR related to hemodynamics, change over the cardiac cycle. Additionally, studies on the association between irregular pulsation and rupture risk have solely focused on static morphological parameters (16–19), while little attention has been given to the connection between changes in morphological characteristics and irregular pulsation.

Consequently, this study aimed to quantify the variations in morphological risk factors over the cardiac cycle in UIAs using 4D-CTA and analyze their correlations with irregular pulsation, which may help clarify the clinical importance of the dynamic change in aneurysms during the cardiac cycle by 4D-CTA.

## Materials and methods

### Patients

A total of 11 patients with 14 UIAs diagnosed by DSA and subsequently examined by 4D-CTA between July 2016 and September 2018 were selected for this study. The Institutional Review Board of Changhai Hospital approved this retrospective study. The requirement for written informed consent was waived and the patients’ information was anonymized and de-identified before analysis.

### 4D-CTA data acquisition

Electrocardiogram (ECG)-gated CTA scanning was performed using a 320-row CT scanner (Aquilion ONE, Toshiba, Japan) in volumetric imaging mode. Primarily, a test bolus was done using a 10-mL non-ionic contrast medium injected at a rate of 5 mL/s to determine the delay time. Subsequently, a 50 mL iodinated contrast medium was administrated intravenously at an infusion rate of 5 mL/s, plus 20 mL saline. 4D-CTA scanning was then started after an appropriate delay time based on the actual situation of each injection test. The scan parameters were: 120-kV tube voltage, 260-mA tube current, the gantry rotation speed of 0.275 s per turn, and z-coverage of 160 mm. The scan time was at least one heartbeat and the in-plane resolution was 0.31 mm.

The source data for 4D-CTA were transferred to an image processing workstation (Vitrea 2 software 6.02, Toshiba, Japan) for ECG-gated reconstruction of the cardiac cycle. The R-R intervals were divided into 20-time phases at 5% intervals to generate 20 sets of CT data packages (image matrix size 512 × 512, slice thickness 0.5 mm), which were saved in DICOM format files.

### Criteria for determining irregular pulsation in IAs

Irregular pulsation on the aneurysmal wall is defined as a focal protuberance, observed in at least three consecutive frames of the 20 CT images that correspond to the 20 phases in the R-R interval, captured by 4D-CTA (11, 20). The determination of IAs with irregular pulsation was made by a neuroradiologist and a neurosurgeon after reviewing 4D-CTA images with concordant results.

### Reconstruction of 3D models of patient-specific IAs

Each DICOM file was analyzed using Mimics software (Materialise Inc.) to extract the aneurysm surface model corresponding to each time phase of the cardiac cycle. To obtain an original 3D aneurysm model, a threshold of cerebrovascular tissue was determined to separate the aneurysm from other tissues such as the skull. In addition, unnecessary small branching arteries were eliminated, the region of interest was segmented using the crop mask, and finally, the aneurysm model was generated by 3D calculation. The resulting 3D structure was exported in STL format to measure morphological characteristics (21, 22) and their cardiac cycle-related variations. The 3D IAs models over a cardiac cycle were segmented independently by Operator 1 using the imaging of each signal source to avoid any bias at best.

### Measurement of morphological characteristics

The 3D aneurysm models were viewed in Geomagic Studio 15.0 (Geomagic Inc.) to determine the neck plane (i.e., the point at which the aneurysm sac protrudes from the parent vessel). Notably, the same uniform neck plane was used for all 20 aneurysm models from the same patient corresponding to the 20-time phases of the cardiac cycle. Each model was divided into five parts, including the aneurysm dome, inlet, outlet, parent artery, and vessels. These parts were imported into MATLAB 7.0 (MathWorks, Natick, Massachusetts, United States) to calculate the morphological parameters (23).

### Aneurysm morphological characteristics

A total of fourteen aneurysm morphological characteristics were included in this study (9, 23, 24), including the maximum diameter of the aneurysm (Dmax), aneurysm height (H), aneurysm width (W), aneurysm surface area (S), aneurysm volume, neck width (NW), the average diameter of parent arteries (Dv), height-to-width ratio (HW), dome-to-neck ratio (DN), bottleneck factor (BNF), AR, SR, nonsphericity index (NSI), and inflow angle. Definitions of aneurysm morphological characteristics are provided in Online Supplement 1 and Online Figure 1.

### Quantification of cardiac cycle-related changes in morphological characteristics

Based on the definitions of the expansion volume $\left(V_{\text{max}}-V_{\text{min}}\right)$ and expansion rate $\left(\frac{V_{\text{max}}-V_{\text{min}}}{V_{\text{min}}}\right)$ during the cardiac cycle proposed by Kuroda et al. (15), we applied this quantification method to other morphological features. Thus, changes in aneurysm morphological characteristics over the cardiac cycle were quantified by (1) absolute change (*), which is the difference between the maximum and minimum values of a parameter over the cardiac cycle, and (2) relative rate of change (%), which is the ratio of the difference between the maximum and minimum values to the minimum value of a parameter over the cardiac cycle.

### Rupture risk assessment

The PHASES score and Juvela score were used to assess the risk of rupture for each UIA. The PHASES score consists of six metrics: population, hypertension, age, size, history of early subarachnoid hemorrhage, and location (6). As the population-based index does not include a score for the Chinese population, we have assumed that they have the same score as the North American and European populations (24). The Juvela score is determined by four indicators: age, cigarette smoking, aneurysm site, and aneurysm size (25).

### Agreement test

The agreement test covered two parts. On the one hand, Operator 1 and Operator 2 independently completed the 3D reconstruction of 5 aneurysms randomly selected from the 14 UIAs 3 months later. The intra- and inter-observers agreements were analyzed by calculating intra-class correlation coefficients (ICCs), to assess the reproducibility of 3D reconstruction in quantifying dynamic changes of aneurysms, which were good (ICC > 0.8). On the other hand, the 3D reconstruction of the 14 UIAs included in this study was performed by Operator 2, who was not aware of the details of Operator 1’s work. Then, the relationships between aneurysm size and morphological variations were analyzed. The details are shown in Online Table 1 and Online Figure 2, and the results showed good agreements between different operators, which can support the reliability of 3D reconstruction in analyzing morphological changes of UIAs.

### Statistical analysis

Statistical analysis was conducted with IBM SPSS Statistics 26 software. To determine the normality of the parameters in the study, the Shapiro–Wilk test was utilized. Based on whether the indicators obeyed normal distribution, parameters were presented as mean ± standard deviation (SD) or median values (range). The Pearson correlation analysis or the Spearman correlation analysis method was used to conduct linear correlation analysis. The rank correlation coefficient (r) ranges from − 1 to + 1, where closer to 0 indicates a weaker correlation and closer to 1 indicates a stronger correlation. The correlations between irregular pulsation and cardiac cycle-related variabilities in morphological characteristics were analyzed by the independent samples t-test or Mann–Whitney U test. The correlations between aneurysm size and cardiac cycle-related changes in morphological characteristics were analyzed by the One-way ANOVA or Kruskal-Wallis H test. Set the significance level as α = 0.05 and consider P< 0.05 (two-tailed) as statistically significant.

## Results

**Figure 1:**
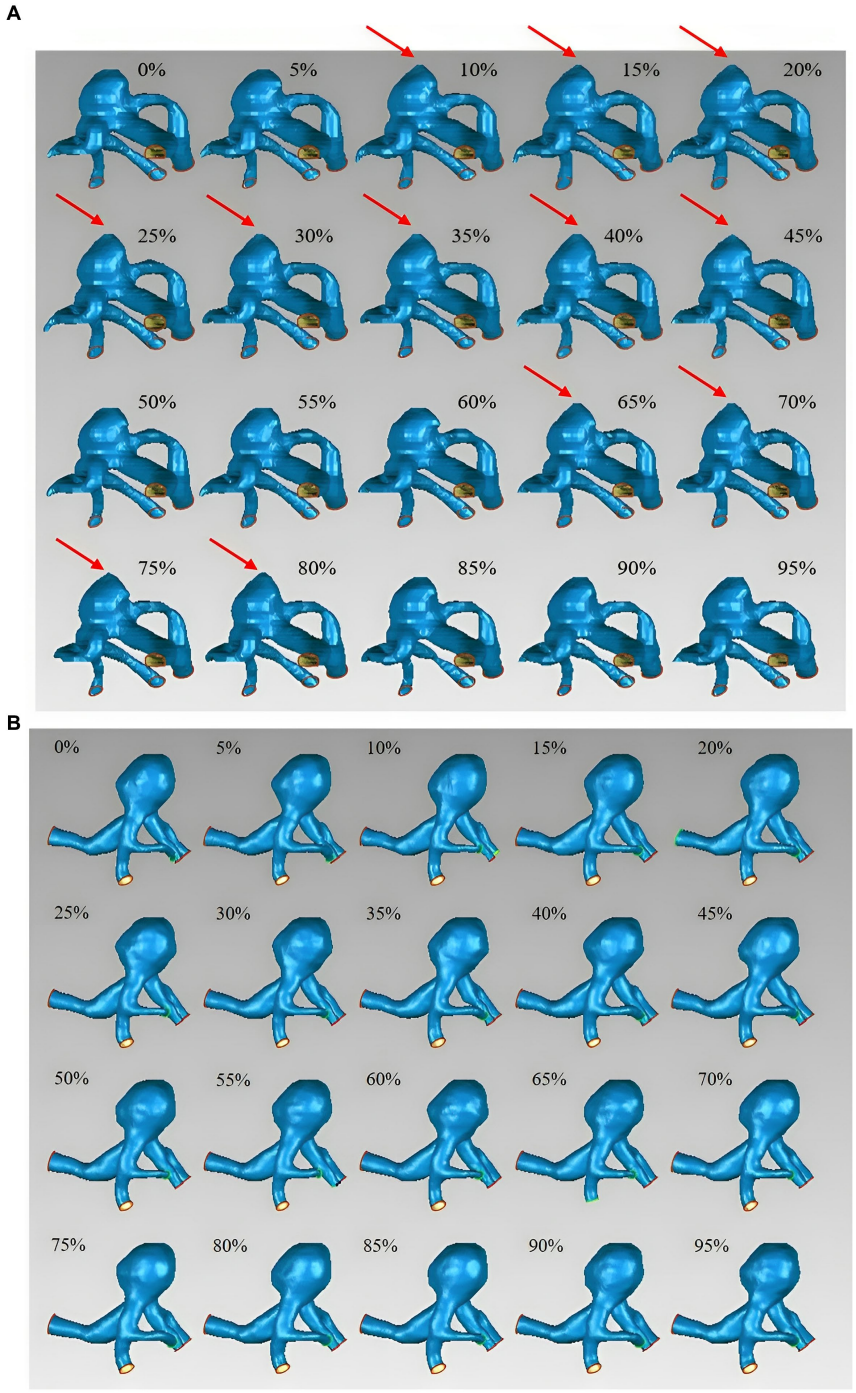
Aneurysm wall motion during the cardiac cycle. **(A)** An aneurysm with focal irregular pulsation during the cardiac cycle (red arrows indicate the presence of irregular pulsation at the aneurysm dome). **(B)** An aneurysm with global dilation during the cardiac cycle.

The basic characteristics of the 11 patients with 14 UIAs included in this study are shown in Table 1. Seven aneurysms exhibited irregular pulsation (50.0%), and others demonstrated global dilation during the cardiac cycle. Figure 1 illustrates two types of aneurysm dynamic change during a cardiac cycle: one with irregular pulsation (Figure 1A) and another with global dilatation (Figure 1B).

**Table 1:** Basic characteristics of the patients and aneurysms.

Patients (No.)	Aneurysm (No.)	Age (Year)	Gender	Location	Dmax (mm)	PHASES score	Juvela score	Irregular pulsation
1	1	52	M	MCA	9.30 ± 0.08	6	5	0
2	2	71	F	MCA	6.55 ± 0.18	4	0	1
3	3	60	F	AComA	3.59 ± 0.07	5	5	0
4	4	72	M	MCA	6.32 ± 0.08	4	2	0
5	5	76	F	MCA	4.56 ± 0.14	4	2	1
6	6	69	F	ICA	16.50 ± 0.14	7	7	0
	7			PComA	7.72 ± 0.06	8	5	0
7	8	71	F	MCA	13.85 ± 0.11	10	3	0
8	9	49	F	VA	3.94 ± 0.14	4	0	1
9	10	64	M	VA	8.48 ± 0.25	8	2	1
10	11	38	M	VA	13.93 ± 0.27	10	7	1
	12			VA	6.41 ± 0.17	4	4	1
11	13	63	F	ICA	7.78 ± 0.03	4	7	0
	14			PComA	5.73 ± 0.18	5	2	1

M, male; F, female. MCA, middle cerebral artery; ICA, internal carotid artery; PComA, posterior communicating artery; AComA = anterior communicating artery; VA, vertebral artery. Dmax, aneurysm maximum diameter. 1 = yes (indicating aneurysms with irregular pulsation); 0 = no (indicating aneurysms with global dilation).

The 14 UIAs were divided into two groups based on the presence or absence of irregular pulsation during the cardiac cycle to investigate the differences in rupture-related morphological parameters, morphological variations over a cardiac cycle, and rupture risk scores between the two groups. The parameters with significant differences are displayed in Table 2, and a complete comparison of the differences between the two groups is provided in Online Table 2.

**Table 2:** The significant differences in morphological parameters between aneurysms with irregular pulsation and global dilation.

Group	Irregular pulsation (*N* = 7)	Global dilation (*N* = 7)	*p*
Parameters			
Dmax* (mm)	0.64 ± 0.16	0.29 ± 0.13	0.001
Dmax% (%)	10.22 ± 2.12	3.62 ± 1.86	< 0.001
H% (%)	8.38 ± 3.08	4.45 ± 2.65	0.025
S% (%)	10.04 (6.23, 22.46)	3.96 (3.11, 7.36)	0.002
V% (%)	13.72 (10.44, 30.67)	6.36 (3.98, 11.49)	0.002
DN% (%)	10.68 (6.52, 23.95)	5.95 (3.23, 8.87)	0.004
AR	0.67 ± 0.17	1.00 ± 0.35	0.042
AR% (%)	10.67 ± 4.40	6.22 ± 3.12	0.050
SR% (%)	13.72 (8.49, 24.58)	7.13 (5.03, 14.25)	0.007
NSI*	0.03 (0.01, 0.05)	0.01 (0.01, 0.01)	0.004
NSI% (%)	29.70 (21.88, 61.90)	11.14 (5.39, 19.98)	0.001

Continuous values conforming to a normal distribution are expressed as mean ± SD, whereas those that deviate from a normal distribution are represented by the median (range). Dmax, aneurysm maximum diameter; H, aneurysm height; S, aneurysm surface area; V, aneurysm volume; DN, the dome-to-neck ratio; AR, aspect ratio; SR, size ratio; NSI, non-sphericity index (parameter)* is the difference between the maximum and minimum values of a parameter in one cardiac cycle; (parameter)% is the ratio of the difference between the maximum value and the minimum value to the minimum value of a parameter in one cardiac cycle.

### Differences in rupture-related morphological parameters between UIAs with irregular pulsation and global dilation

Aneurysm maximum diameter (7.09 ± 3.36 mm vs. 9.29 ± 4.45 mm, *p* = 0.315), volume (74.96 mm^3^ vs. 178.42 mm^3^, *p* = 0.165), and location (*p* = 0.073) were not significantly different between the two groups. Only a significant difference was observed in AR between the two groups (0.67 ± 0.17 vs. 1.00 ± 0.35, *p* = 0.042). In addition, there were no significant differences between irregular pulsation and rupture risk scores (PHASES score, *p* = 0.423; Juvela score, *p* = 0.058).

### Differences in cardiac cycle-related morphological variations between UIAs with irregular pulsation and global dilation

The absolute changes and relative rates of change in aneurysm maximum diameter (0.64 ± 0.16 mm vs. 0.29 ± 0.13 mm, *p* = 0.001; 10.22% ± 2.12% vs. 3.62% ± 1.86%, P < 0.001), as well as in NSI (0.03 vs. 0.01, *p* = 0.004; 29.70% vs. 11.14%, p = 0.001) were significantly different between the two groups. Besides, the relative change rates in aneurysm height (8.38% ± 3.08% vs. 4.45% ± 2.65%, *p* = 0.025), surface area (10.04% vs. 3.96%, *p* = 0.002), volume (13.72% vs. 6.36%, p = 0.002), DN (10.68% vs. 5.95%, p = 0.004), AR (10.67% ± 4.40% vs. 6.22% ± 3.12%, *p* = 0.050), and SR (13.72% vs. 7.13%, *p* = 0.007) were also significantly different between the two groups.

Of the 14 UIAs included in this study, there were seven small aneurysms (Dmax < 7 mm), four large aneurysms (7 mm ≤ Dmax < 10 mm), and three giant aneurysms (Dmax ≥ 10 mm). It is worth noting that out of the 7 UIAs with irregular pulsation, 5 were small aneurysms (71.43%). In contrast, only 2 of the 7 UIAs with global dilatation were small aneurysms (28.57%). Hence, the 14 UIAs were further divided into three groups based on the aneurysm size, and the differences in morphological changes among the three groups were analyzed.

### Correlations between aneurysm size and the changes during the cardiac cycle

**Figure 2:**
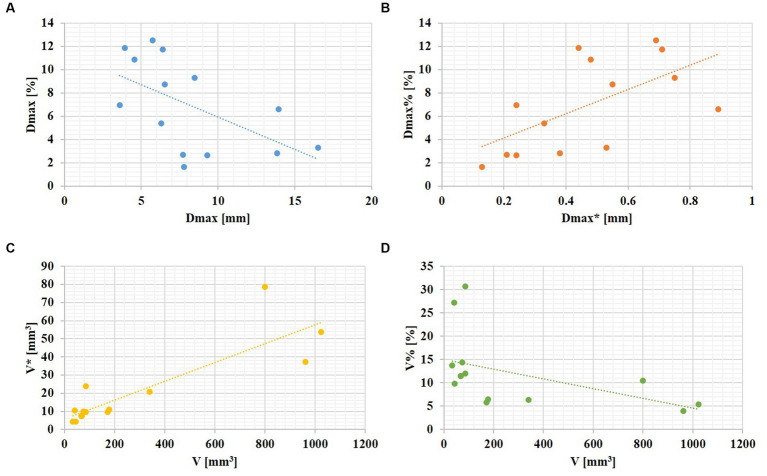
The linear relationships between aneurysm size and its changes over the cardiac cycle. **(A)** An aneurysm with focal irregular pulsation during the cardiac cycle (red arrows indicate the presence of irregular pulsation at the aneurysm dome). **(B)** An aneurysm with global dilation during the cardiac cycle.

Aneurysm maximum diameter was negatively associated with its relative change rate (r = − 0.561, *p* = 0.037). In addition, aneurysm volume was highly positively correlated with its absolute change (r = 0.837, P < 0.001), and negatively correlated with its relative change rate (r = − 0.688, *p* = 0.007). The linear relationships are shown in Figure 2.

### Correlations between aneurysm size and cardiac cycle-related changes in morphological characteristics

**Figure 3:**
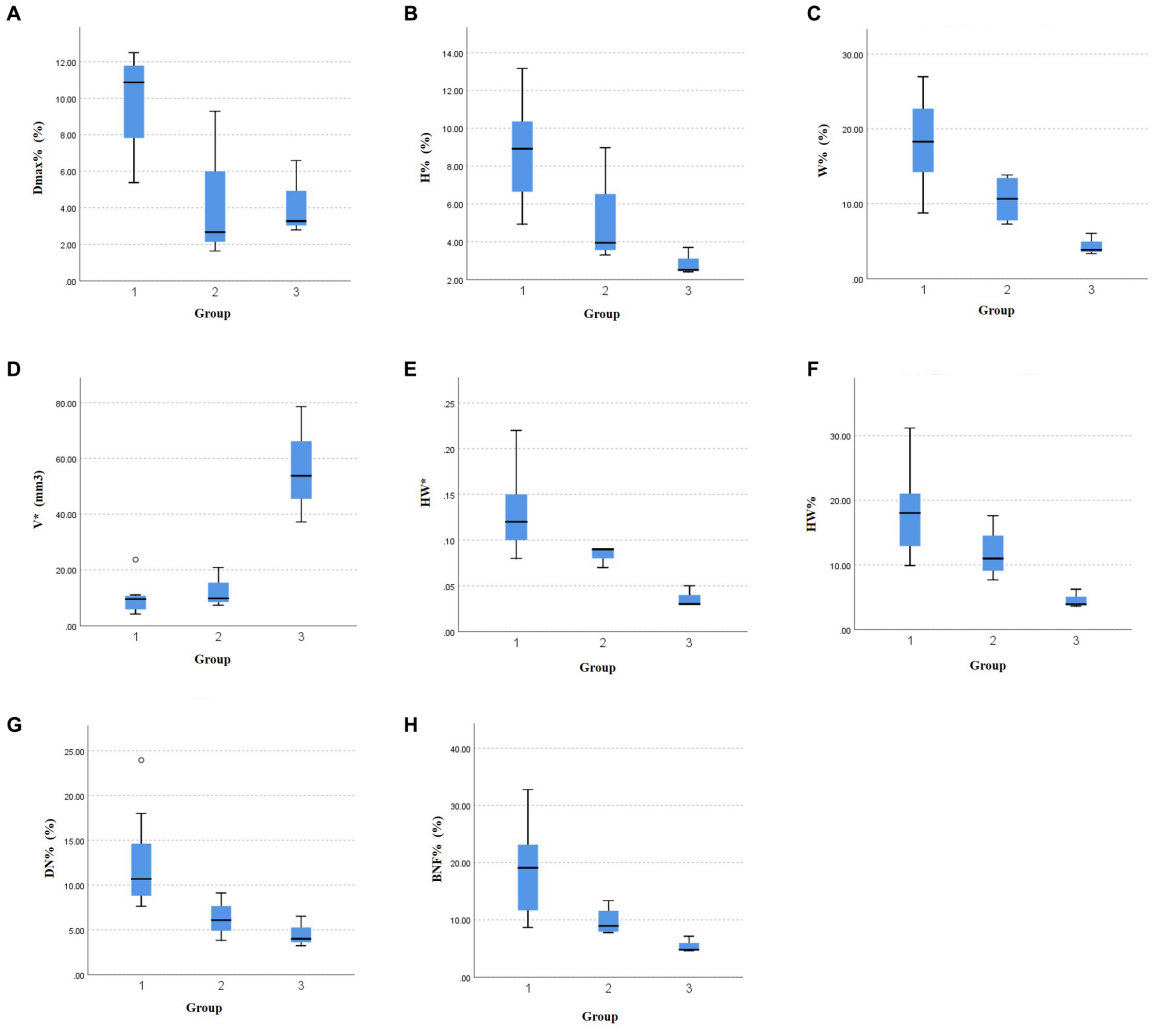
The significant relationships between aneurysm size and morphological variations during the cardiac cycle. The differences in the relative change rate of **(A)** the maximum diameter, **(B)** aneurysm height, **(C)** aneurysm width, **(F)** height-to-width ratio, **(G)** dome-to-neck ratio, and **(H)** bottleneck factor, as well as the absolute change of **(D)** aneurysm volume, **(E)** height-to-width ratio among the three groups.

Table 3 displays the variations in morphological changes among the three groups of aneurysms with different sizes, while Figure 3 illustrates the parameters with significant correlations. The absolute change in aneurysm volume (*p* = 0.035) and HW (*p* = 0.009) were significantly different among the three groups. The relative rates of change in aneurysm maximum diameter (*p* = 0.013), height (*p* = 0.014), width (*p* = 0.008), HW (*p* = 0.020), DN (*p* = 0.019), and BNF (*p* = 0.012) were also significantly different among the three groups.

**Table 3:** Correlations between aneurysm size and cardiac cycle-related changes in morphological characteristics.

Group	Small aneurysms (*N* = 7)	Large aneurysms (*N* = 4)	Giant aneurysms (*N* = 3)	*p*
Dmax* (mm)	0.49 ± 0.17	0.33 ± 0.28	0.60 ± 0.26	0.318
Dmax% (%)	9.71 ± 2.75	4.06 ± 3.52	4.22 ± 2.07	**0.013**
H* (mm)	0.34 ± 0.12	0.26 ± 0.06	0.29 ± 0.03	0.564
H% (%)	8.72 ± 2.86	5.04 ± 2.64	2.87 ± 0.72	**0.014**
W* (mm)	0.81 ± 0.21	0.74 ± 0.31	0.56 ± 0.25	0.381
W% (%)	18.27 ± 6.70	10.61 ± 3.32	4.41 ± 1.45	**0.008**
S* (mm^2^)	7.77 ± 4.53	6.07 ± 1.01	17.07 ± 4.46	0.051
S% (%)	11.93 ± 6.69	6.19 ± 3.11	4.39 ± 1.64	0.054
V* (mm^3^)	10.10 ± 6.61	11.97 ± 6.07	56.53 ± 20.81	**0.035**
V% (%)	15.89 ± 9.24	9.52 ± 4.16	6.60 ± 3.40	0.093
NW* (mm)	0.42 ± 0.25	0.4 ± 0.06	0.46 ± 0.15	0.523
NW% (%)	7.64 ± 4.96	5.86 ± 2.29	4.34 ± 2.00	0.433
Dv* (mm)	0.29 ± 0.18	0.2 ± 0.07	0.32 ± 0.13	0.413
Dv% (%)	11.86 ± 7.71	7.11 ± 3.35	11.29 ± 7.20	0.697
HW*	0.13 ± 0.05	0.09 ± 0.01	0.04 ± 0.01	**0.009**
HW% (%)	18.14 ± 7.40	11.81 ± 4.19	4.58 ± 1.43	**0.020**
DN*	0.06 ± 0.04	0.05 ± 0.04	0.03 ± 0.03	0.594
DN% (%)	12.73 ± 6.01	6.27 ± 2.17	4.58 ± 1.72	**0.019**
BNF*	0.15 ± 0.07	0.10 ± 0.03	0.06 ± 0.02	0.068
BNF% (%)	18.58 ± 8.56	9.75 ± 2.56	5.51 ± 1.42	**0.012**
AR*	0.07 ± 0.03	0.06 ± 0.04	0.05 ± 0.03	0.554
AR% (%)	10.65 ± 3.80	7.46 ± 4.83	4.63 ± 1.71	0.107
SR*	0.26 ± 0.13	0.17 ± 0.07	0.44 ± 0.25	0.161
SR% (%)	14.91 ± 5.52	6.98 ± 1.24	11.91 ± 7.52	0.071
NSI*	0.03 ± 0.02	0.01 ± 0.00	0.01 ± 0.0	0.064
NSI% (%)	37.33 ± 21.07	15.62 ± 10.10	13.04 ± 9.03	0.077
Inflow Angle ( ° )	118.12 ± 53.50	107.61 ± 57.53	118.42 ± 17.96	0.939

Dmax, aneurysm maximum diameter; H, aneurysm height; W, aneurysm width; S, aneurysm surface area; V, aneurysm volume; NW, neck width; Dv, the average diameter of parent arteries; HW, the height-to-width ratio; DN, the dome-to-neck ratio; BNF, the bottleneck factor; AR, aspect ratio; SR, size ratio; NSI, nonsphericity index. Values are expressed as mean ± SD. The bold values indicate statistically significant differences in the parameters among the three groups.

## Discussion

To our knowledge, the dearth of quantitative analysis regarding cardiac cycle-related dynamic changes in UIAs and their correlation with aneurysm rupture risk limits the accuracy of rupture risk assessment and the clinical relevance of irregular pulsation by 4D-CTA. In the present study, we quantified the dynamic changes in morphological characteristics and compared their associations with irregular pulsation of 14 UIAs during the cardiac cycle. In the present study, we discovered that the smaller the UIAs, the more dramatic the changes in aneurysm morphological characteristics during the cardiac cycle. Moreover, irregular pulsation was significantly associated with greater variations in aneurysm size (especially in longitudinal length) and shape. This proof-of-concept study could help to explain the importance of dynamic changes using 4D-CTA in assessing the rupture risk of UIAs.

Kuroda et al. (15) reported an interobserver variation of 11.9 ± 17.6 mm^3^ in IAs and 1.54 ± 3.90 mm^3^ in normal arteries. They also suggested that a volume change of 0.248% should be considered an insignificant artifact for IAs during a cardiac cycle. In the present study, we performed an agreement test which showed good intra- and inter-agreements (ICC > 0.8). The results confirmed the reproducibility of 3D aneurysm reconstruction in quantifying morphological variations during a cardiac cycle.

Intracranial aneurysm wall motion is a potential predictor for aneurysm rupture (26). The reliability of capturing aneurysm wall motion varies among imaging techniques. Although DSA is the gold standard for assessing IAs due to its superior spatial resolution, its invasive nature has restricted an extensive clinical utility. Multi-slice CT with shuttle or toggling table modes can be used to achieve 4D-CT data acquisition by moving the scan bed, but motion artifacts are unavoidable (12). The 320-row detector CT enables 4D-CT data acquisition in volume mode (4D-CTA). The wide coverage allows fast scanning of patients without moving the scan bed, greatly reducing the effects of motion artifacts. The reliability of 4D-CTA to observe aneurysm irregular pulsation has been initially validated (27). Under 4D-CTA, the incidence of irregular pulsation ranges from 26.42 to 40.63% in UIAs (20, 28–30), and 17.39–83.33% in RIAs (17, 28). The present study found irregular pulsation in 7 out of 14 UIAs (50.00%) by 4D-CTA.

Aneurysm size, volume, and location were not significantly associated with irregular pulsation in this study. Aneurysm size (maximum diameter, Dmax) is the most frequently used parameter to evaluate aneurysm rupture risk in clinical practice, as larger UIAs have higher rupture risks (7). Nevertheless, assessing aneurysm rupture risk by considering only the aneurysm size is not sufficiently accurate because most ruptured aneurysms are small (partly due to their high incidence) (31). In the study by Zhou et al. (17) that included 217 small IAs (14 RIAs), they found that the incidence of irregular pulsation was 35.5%. Gu et al. included 168 small IAs (102 RIAs, and 66 UIAs) and reported that irregular pulsation occurred in 23 of 66 UIAs (34.85%). Of the 14 UIAs included in this study, 7 were small aneurysms (Dmax < 7 mm), 5 of which exhibited irregular pulsation during the cardiac cycle by 4D-CTA (71.43%). The high prevalence of irregular pulsation in small UIAs found in the present study may be partly due to the small sample size, but can also support the argument that the risk of rupture for small aneurysms is not negligible.

A significant association was observed between lower AR and irregular pulsation (*p* = 0.042) in this study, which appears to contradict findings from prior studies (16, 18, 32). In addition, there were no significant differences between irregular pulsation and rupture risk scores (PHASES score, *p* = 0.423; Juvela score, *p* = 0.058). The limited sample size and the high proportion of small aneurysms could account for the lack of statistical significance in the results. Beyond that, whether the PHASES score or the Juvela score is not considered other rupture-related factors such as AR, SR, and wall shear stress (WSS), which could underestimate the actual rupture risk.

Nevertheless, aneurysms with irregular pulsation exhibited greater degrees of changes in aneurysm size, height, volume, DN, AR, SR, and NSI in the present study. AR and SR are risk factors for aneurysm rupture due to their impact on hemodynamics. An increased AR is associated with localized and slow flow in the aneurysm dome (33). SR measures the maximum deformation of the parent vessel due to the outpouching of an aneurysm, as a large SR leads to complex flow patterns, multiple vortices, and low WSS (9, 23). In this study, the maximum diameter of most aneurysms was in the longitudinal direction, indicating direct exposure of the aneurysmal dome to dynamic blood flow during the cardiac cycle. Hence, the top of the aneurysm is a common site of rupture due to the direct impact of blood flow (13, 34). Significant changes were found in the longitudinal morphologies such as the maximum diameter and height, in contrast to the transverse parameters such as aneurysm width and neck width. This ultimately caused substantial variations in both aneurysm size and shape. This suggests that a smaller maximum diameter of the UIA may result in a more pronounced change in length in this direction over the cardiac cycle, which may increase the likelihood of focal irregular pulsation, especially at the aneurysm dome.

Zhang et al. (35) included 328 IAs (including 37 ruptured IAs, 60 symptomatic, and 231 asymptomatic IAs) and reported that irregular pulsation was an independent risk factor for aneurysm symptomatic and rupture. The results suggest that irregular pulsation observed by 4D-CTA could identify not only RIAs but also symptomatic UIAs, which might help clinical rupture risk stratification for UIAs. However, there are relatively few studies of irregular pulsation in asymptomatic UIAs.

A review by Stam et al. (36) summarized that the absolute change of aneurysm volume ranged from 14 ± 9 mm^3^ to 106 ± 123 mm^3^, and the mean relative change rate of aneurysm volume ranged from 5.4% ± 4.1 to 36.8% ± 9.5% (without limitations in imaging modalities). In this study, the absolute change in aneurysm volume was 4.21–78.58 mm^3^, and the relative rate of change varied from 3.98 to 30.67% over a cardiac cycle by 4D-CTA. Stam et al. also reported that aneurysm size was positively associated with the absolute change of aneurysm volume, which was consistent with the results of this study. We found that the magnitude of change in aneurysm volume over the cardiac cycle increased significantly with larger aneurysm size (*p* = 0.035). We also found despite an increase in pulsation amplitude with increasing aneurysm size, the degree of change was not significant relative to the aneurysm size. Conversely, smaller UIAs caused more noteworthy degrees of change despite the lower amplitude of change. In addition, smaller aneurysms exhibited greater changes in morphological features than larger aneurysms, despite the larger aneurysms having significantly greater volume variations.

## Limitations

Firstly, it is important to note that 4D-CTA is not a commonly used method for routine clinical examination of intracranial aneurysms. Therefore, the small sample size and selective bias of the subjects included in this study were unavoidable and resulted in statistically insignificant outcomes. Secondly, this study exhibits selection bias given it is a single-center retrospective study and the fact that 4D-CTA is not routinely utilized in clinical examinations for IAs. Additionally, a golden standard for quantifying aneurysm pulsation is still lacking. Further research with a larger sample size is necessary to confirm the clinical importance of quantifying changes in aneurysm morphologic parameters during the cardiac cycle.

## Conclusion

Although larger aneurysms experienced significant volume changes, changes in morphologic risk factors were more pronounced in small aneurysms. Furthermore, greater alterations in morphologic risk factors were significantly associated with irregular pulsation, suggesting an increased risk of rupture for small UIAs. However, additional risk factors need to be included in future analyses to explore which poses a higher risk of rupture for small aneurysms with irregular pulsation compared with large aneurysms with global dilation during the cardiac cycle.

## Data availability statement

The raw data supporting the conclusions of this article will be made available by the authors, without undue reservation.

## Ethics statement

The Institutional Review Board of Changhai Hospital approved this retrospective study. The requirement for written informed consent was waived and the patients’ information was anonymized and de-identified before analysis.

## Author contributions

SC: Conceptualization, Data curation, Formal analysis, Investigation, Methodology, Writing – original draft. NL: Conceptualization, Data curation, Resources, Validation, Writing – review & editing. YQ: Validation, Writing – review & editing. MZ: Project administration, Supervision, Writing – review & editing. TZ: Funding acquisition, Project administration, Writing – review & editing. YC: Funding acquisition, Project administration, Supervision, Writing – review & editing.
