# Supplementary material for: Relationships between irregular pulsation and variations in morphological characteristics during the cardiac cycle in unruptured intracranial aneurysms by 4D-CTA
Source: Front Neurol. 2024 Mar 28;15:1302874. doi: 10.3389/fneur.2024.1302874 (PMC11005792; doi:10.3389/fneur.2024.1302874)
Supplement: Supplementary file 1 [file Data_Sheet_1.docx]

### Online Supplement 1 Definitions of morphological characteristics

1. Maximum diameter (Dmax)

The maximum diameter of the aneurysm can be calculated as either the maximum height (the maximum distance from the midpoint of the aneurysm neck plane to any point on the aneurysm dome) or the maximum width (the greatest distance across the aneurysm wall).

1. Aneurysm height (H)

The height of the aneurysm was the perpendicular height, determined as the vertical distance from the dome of the aneurysm to the midpoint of the neck plane.

1. Aneurysm width (W)

The maximum distance perpendicular to the maximum diameter of the aneurysm.

1. Neck width (NW)

The neck width was calculated as twice the mean distance from the midpoint of the neck plane to the wall of the aneurysm.

1. The average diameter of parent arteries (Dv)

The average diameter of parent arteries was calculated separately for sidewall aneurysms or bifurcation aneurysms, shown in Figure 1.

1. Height-to-width ratio (HW)

The ratio of aneurysm height to the aneurysm width.

1. Dome-to-neck ratio (DN)

The ratio of the maximum diameter of the aneurysm to the aneurysm neck width.

1. Bottleneck factor (BNF)

The ratio of the maximum diameter parallel to the neck plane to the width of the aneurysm neck.

1. Aspect ratio (AR)

The ratio of aneurysm height to the average neck diameter.

1. Size ratio (SR)

The ratio of the maximum diameter to the average diameter of parent arteries.

1. Non-sphericity index (NSI)

$$NSI=1-\left( 18\pi\right)^{\frac{1}{3}}V^{\frac{2}{3}}/S$$

in which $V$ and $S$ are the (12) volume and (13) surface area of the aneurysm body, separately. NSI correlates with the ellipticity and surface smoothness of the aneurysm, so it can be viewed as a combination of the ellipticity index (EI) and the undulation index (UI).

(14) Inflow angle

The inflow angle is the angle between the mid-axis of the proximal parent artery and the inclination of the aneurysm.

### Online Figure 1
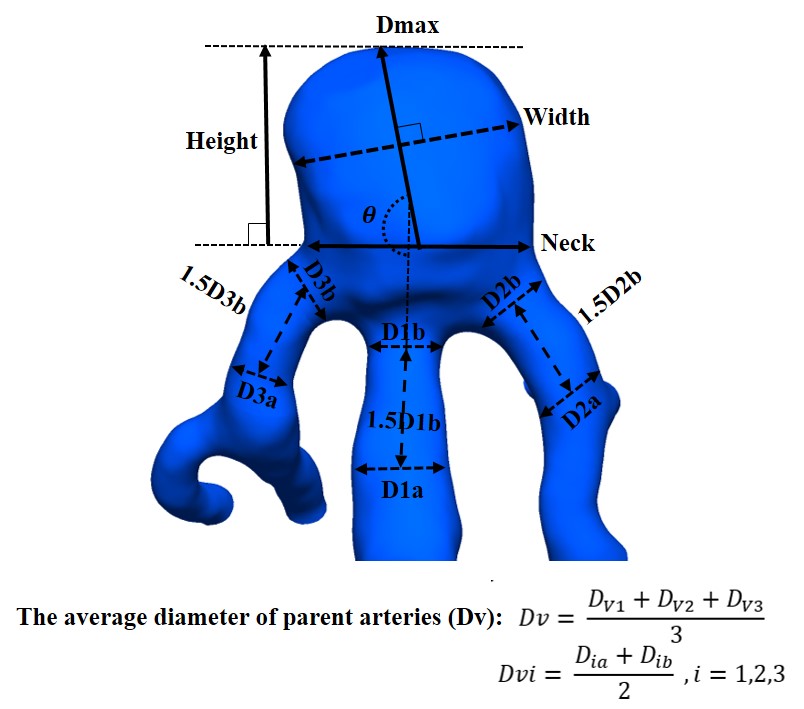
 The morphological characteristics of intracranial aneurysms

### Online Table 1 Agreement test (Correlations between aneurysm size and cardiac cycle-related changes in morphological characteristics)

| Group | Small aneurysms  (N = 7) | Large aneurysms  (N = 4) | Giant aneurysms  (N = 3) | P |
| --- | --- | --- | --- | --- |
| Dmax* (mm) | 0.47$\pm$0.15 | 0.30$\pm$0.23 | 0.56$\pm$0.21 | 0.401 |
| Dmax% (%) | 10.02$\pm$2.90 | 4.20$\pm$3.71 | 4.50$\pm$2.21 | **0.012** |
| H* (mm) | 0.41$\pm$0.18 | 0.30$\pm$0.10 | 0.33$\pm$0.05 | 0.601 |
| H% (%) | 8.86$\pm$2.99 | 5.21$\pm$2.81 | 2.99$\pm$0.80 | **0.007** |
| W* (mm) | 0.90$\pm$0.31 | 0.88$\pm$0.40 | 0.74$\pm$0.27 | 0.405 |
| W% (%) | 18.30$\pm$5.30 | 10.90$\pm$4.20 | 4.14$\pm$1.54 | **0.047** |
| S* (mm2) | 8.00$\pm$4.80 | 7.00$\pm$1.25 | 17.97$\pm$4.76 | **0.025** |
| S% (%) | 12.45$\pm$7.23 | 7.36$\pm$3.91 | 4.40$\pm$1.87 | 0.051 |
| V* (mm3) | 11.34$\pm$6.89 | 13.01$\pm$6.50 | 58.02$\pm$23.22 | **0.037** |
| V% (%) | 15.24$\pm$9.45 | 9.86$\pm$4.04 | 6.30$\pm$3.39 | 0.501 |
| NW* (mm) | 0.41$\pm$0.28 | 0.39$\pm$0.08 | 0.34$\pm$0.15 | 0.490 |
| NW% (%) | 7.80$\pm$4.98 | 5.99$\pm$2.32 | 4.55$\pm$2.27 | 0.501 |
| Dv* (mm) | 0.27$\pm$0.18 | 0.21$\pm$0.11 | 0.35$\pm$0.13 | 0.400 |
| Dv% (%) | 11.90$\pm$7.74 | 7.30$\pm$3.53 | 10.89$\pm$7.23 | 0.699 |
| HW* | 0.13$\pm$0.04 | 0.09$\pm$0.01 | 0.04$\pm$0.01 | **0.012** |
| HW% (%) | 18.10$\pm$7.40 | 11.80$\pm$4.20 | 4.60$\pm$1.44 | **0.023** |
| DN* | 0.06$\pm$0.04 | 0.05$\pm$0.04 | 0.03$\pm$0.03 | 0.600 |
| DN% (%) | 12.88$\pm$6.20 | 6.36$\pm$2.20 | 4.63$\pm$1.75 | **0.018** |
| BNF* | 0.15$\pm$0.06 | 0.10$\pm$0.03 | 0.06$\pm$0.02 | 0.070 |
| BNF% (%) | 18.58$\pm$8.56 | 9.75$\pm$2.56 | 5.51$\pm$1.42 | **0.012** |
| AR* | 0.07$\pm$0.03 | 0.06$\pm$0.03 | 0.05$\pm$0.03 | 0.556 |
| AR% (%) | 10.81$\pm$3.82 | 7.47$\pm$4.85 | 4.61$\pm$1.70 | 0.164 |
| SR* | 0.28$\pm$0.12 | 0.17$\pm$0.06 | 0.45$\pm$0.26 | 0.160 |
| SR% (%) | 14.90$\pm$5.50 | 7.00$\pm$1.25 | 11.90$\pm$7.50 | 0.070 |
| NSI* | 0.02$\pm$0.01 | 0.01$\pm$0.00 | 0.01$\pm$0.00 | 0.060 |
| NSI% (%) | 35.73$\pm$21.55 | 15.80$\pm$10.30 | 13.55$\pm$8.67 | 0.087 |
| Inflow Angle ($\boldsymbol{^{\circ})}$ | 115.01$\pm$50.38 | 109.61$\pm$64.54 | 120.01$\pm$15.04 | 0.805 |

**Note:** Dmax, aneurysm maximum diameter; H, aneurysm height; W, aneurysm width; S, aneurysm surface area; V, aneurysm volume; NW, neck width; Dv, the average diameter of parent arteries; HW, the height-to-width ratio; DN, the dome-to-neck ratio; BNF, the bottleneck factor; AR, aspect ratio; SR, size ratio; NSI, nonsphericity index. Values are expressed as mean $\pm$ SD. The bold values indicate the statistically significant differences in the parameters among the three groups.

**Online Figure 2 The significant relationships between aneurysm size and morphological variations during the cardiac cycle**


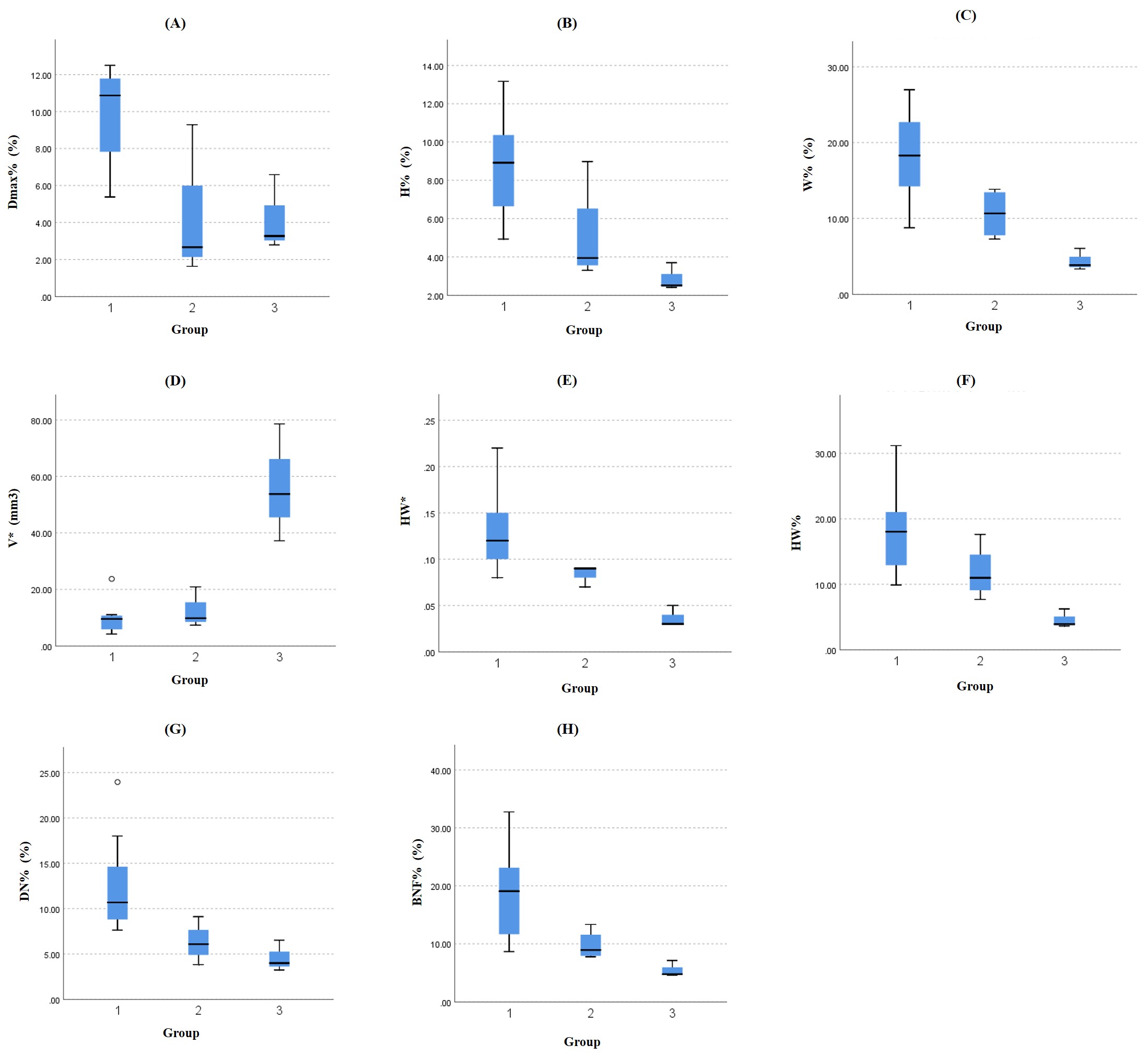

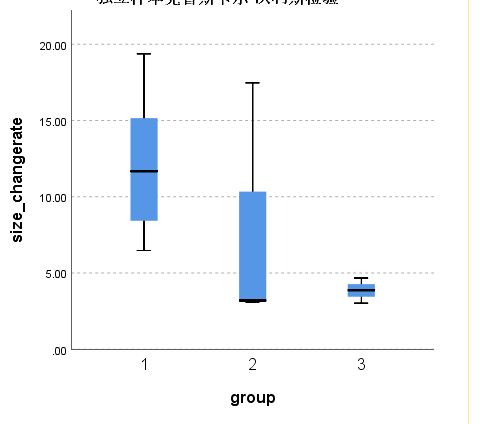

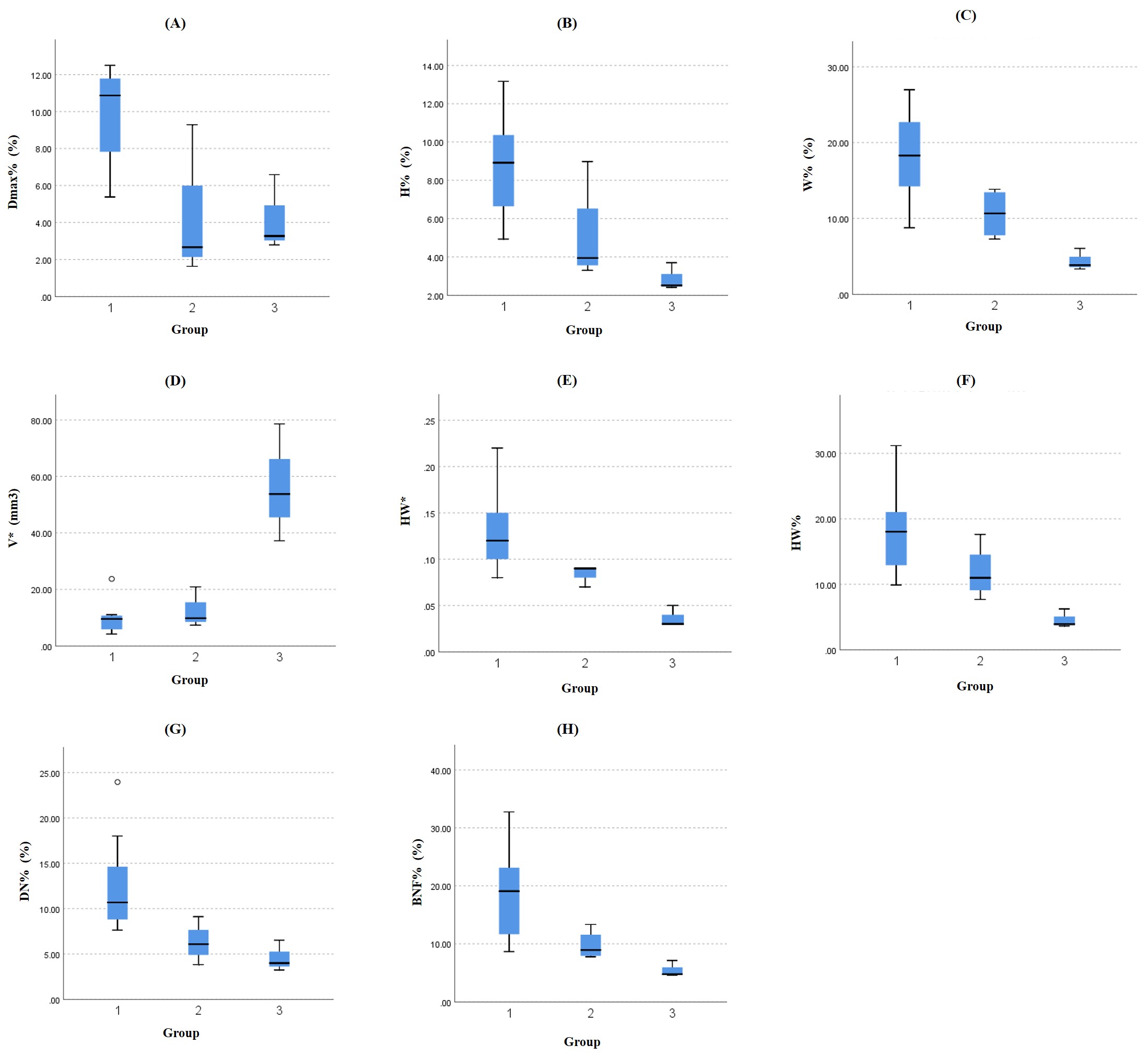

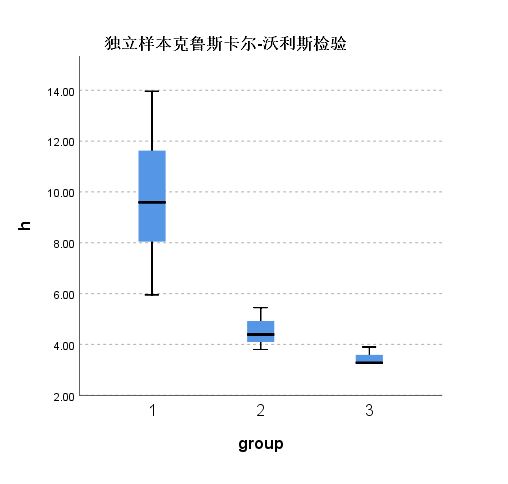

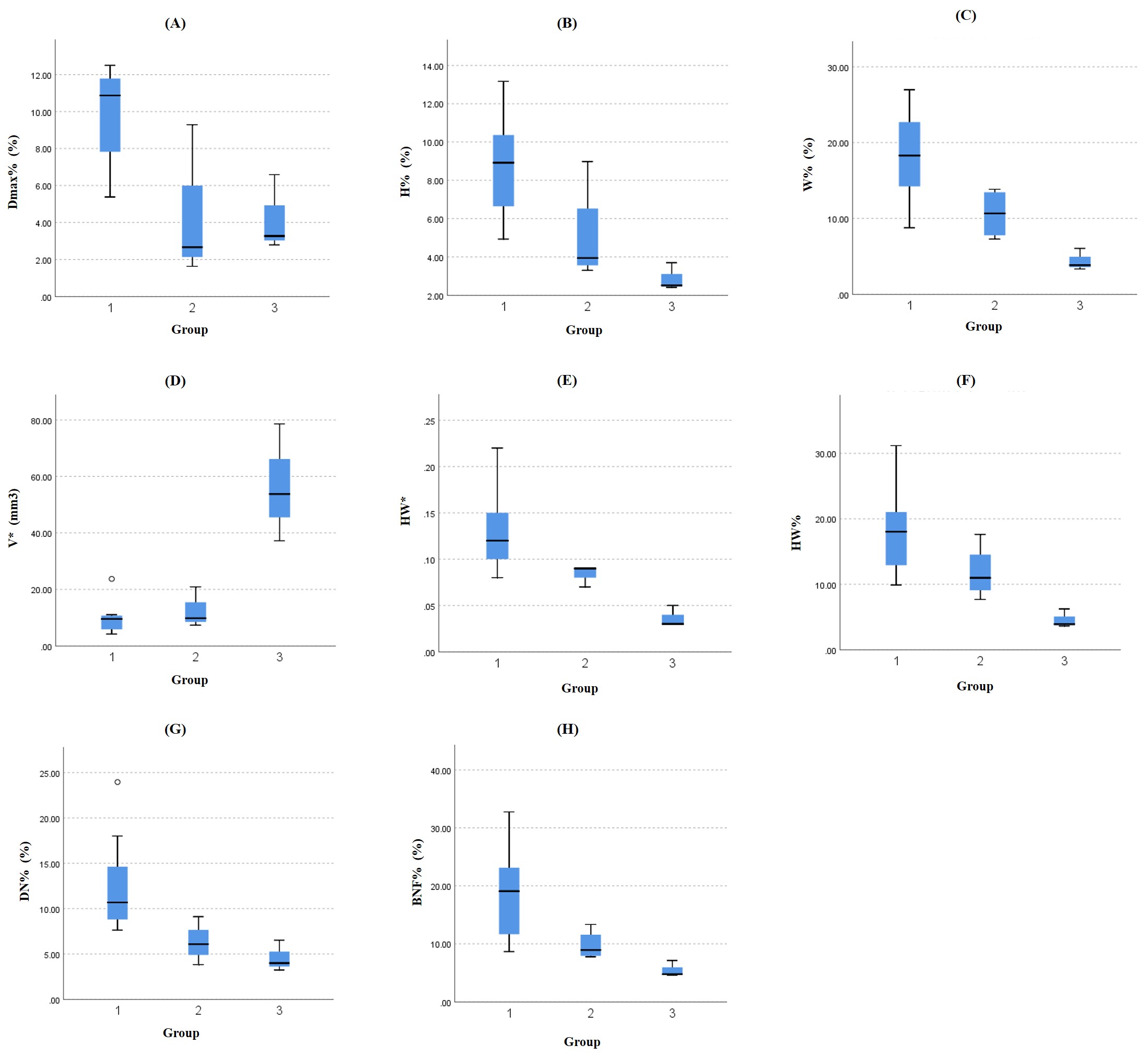

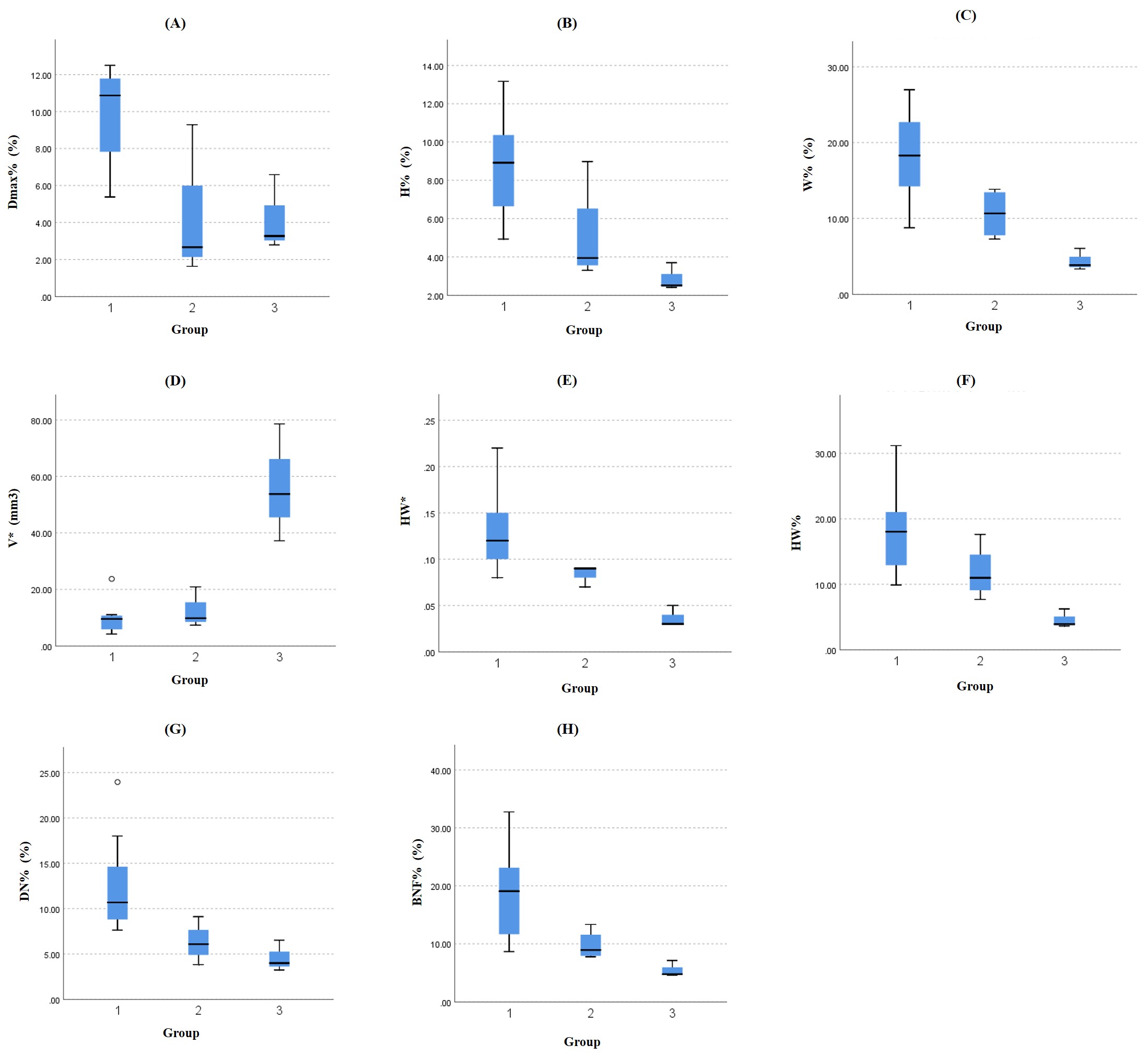

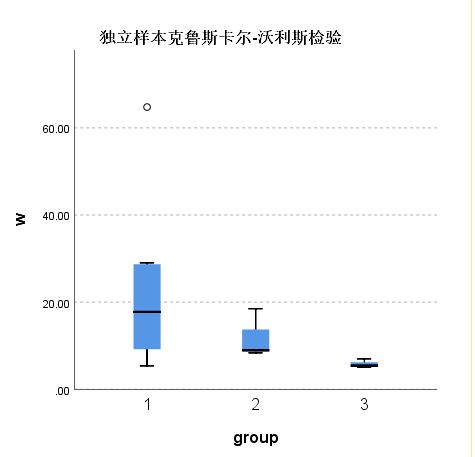

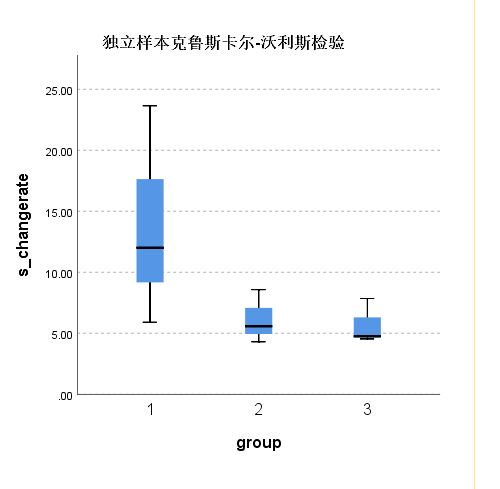


**S% (%)**


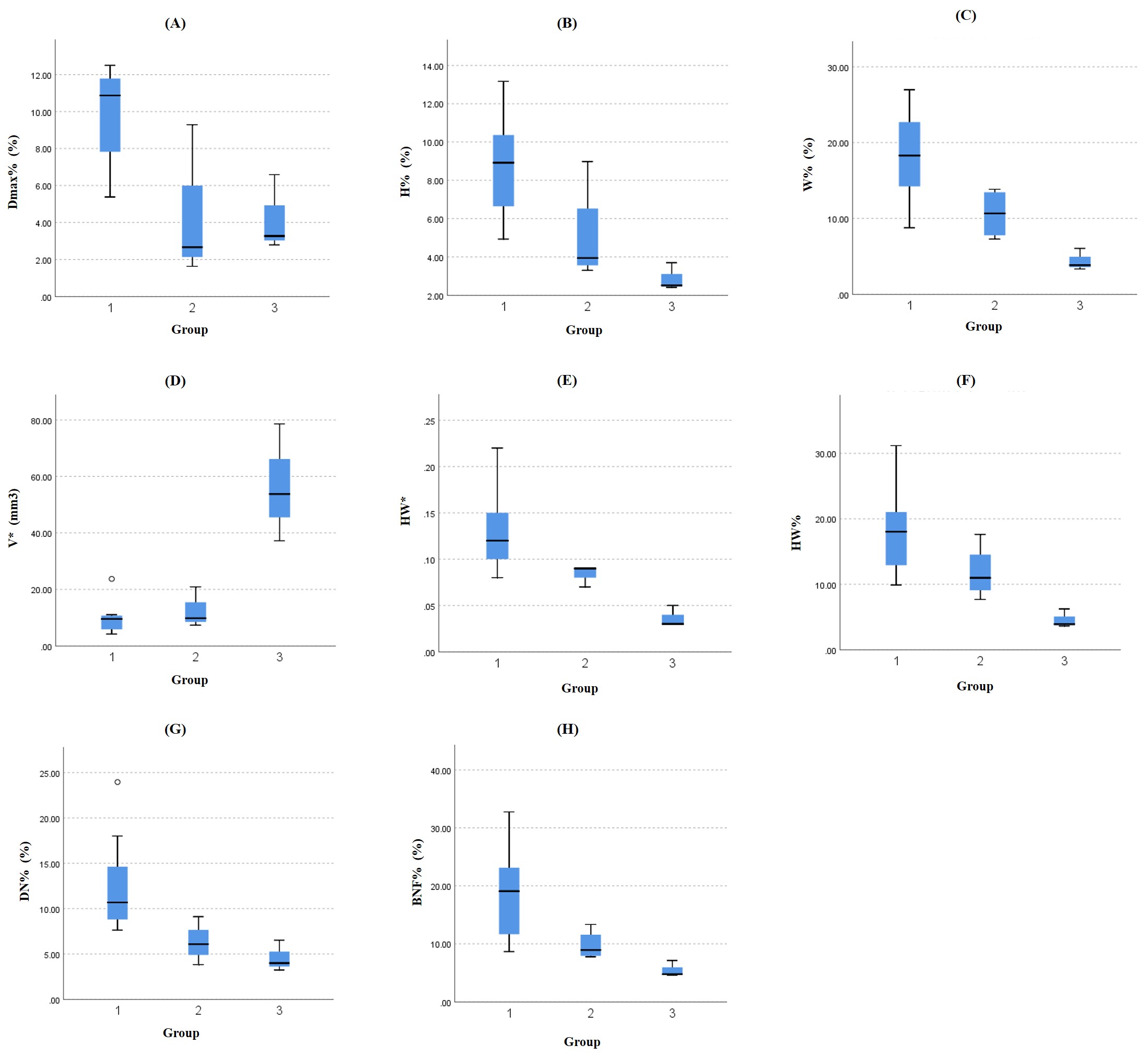

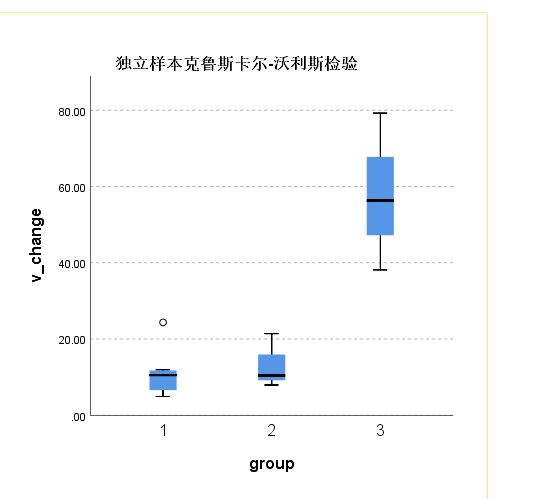

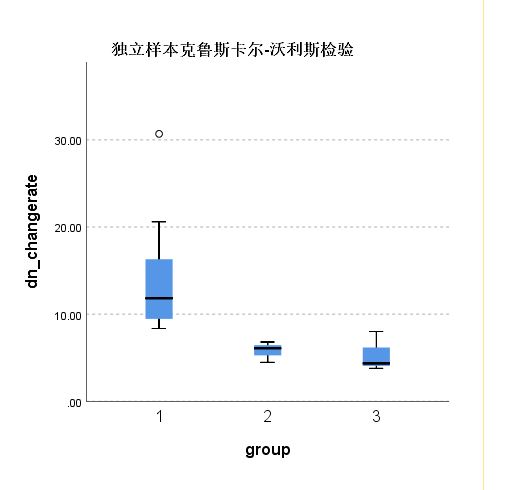

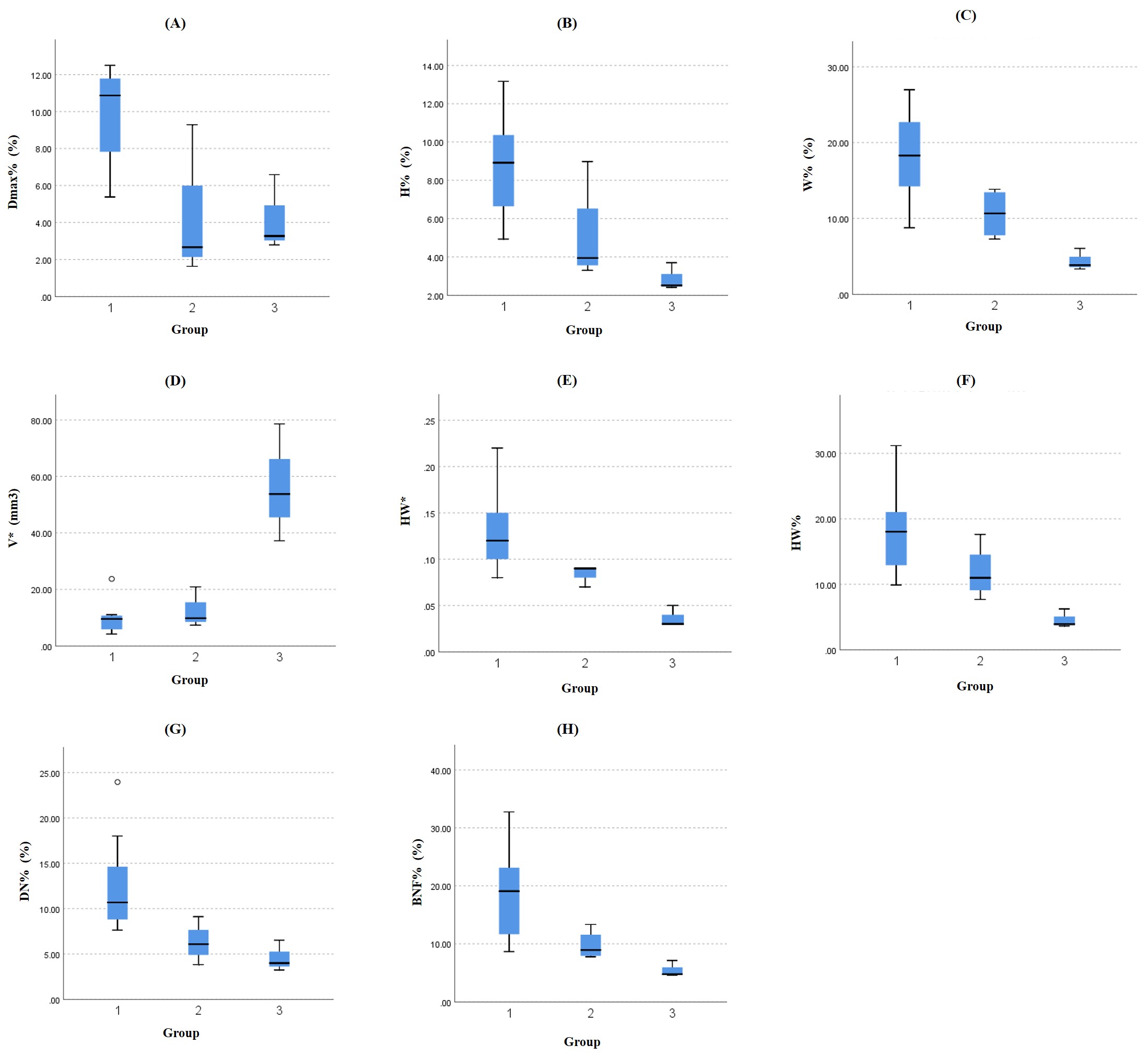

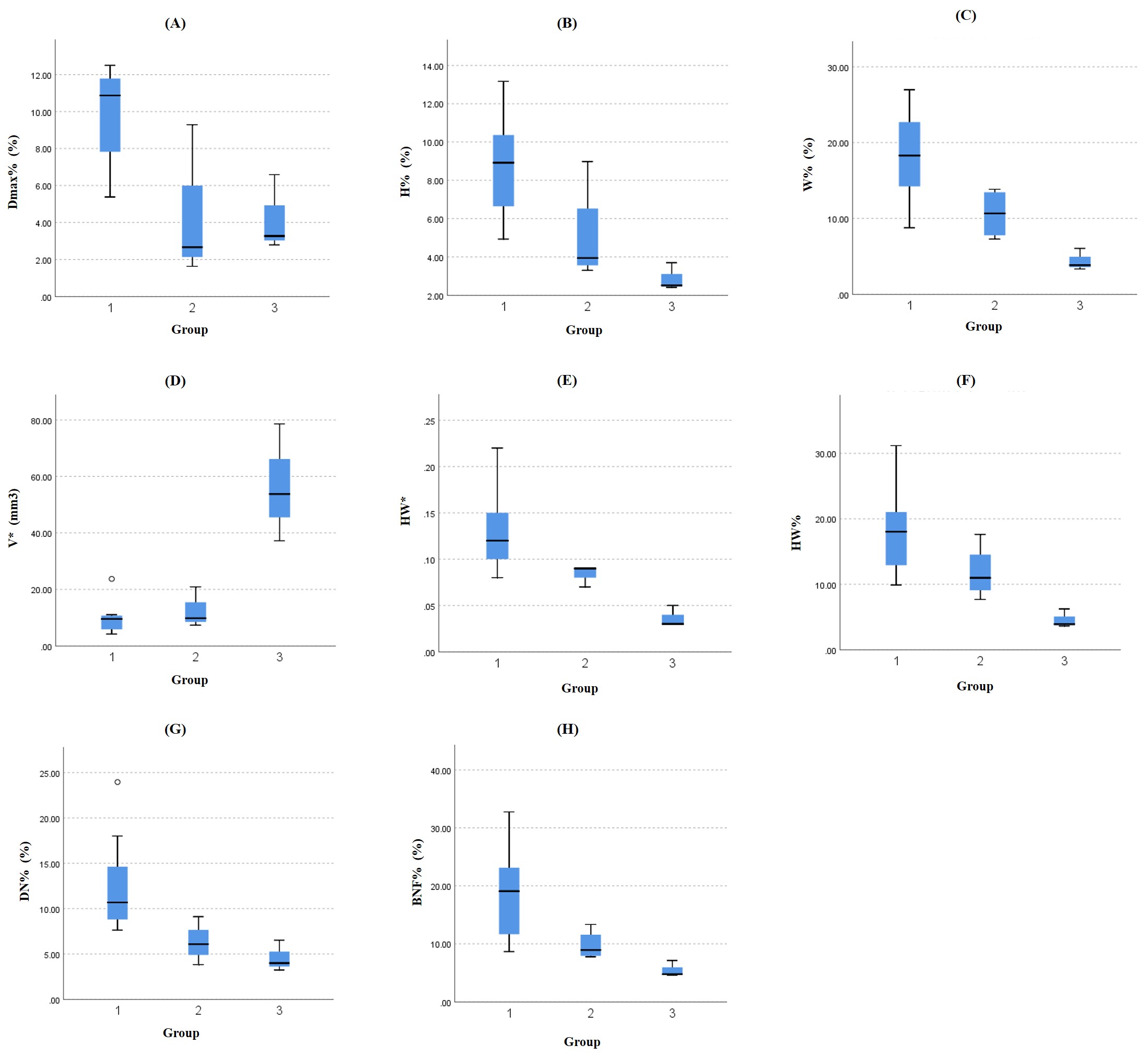

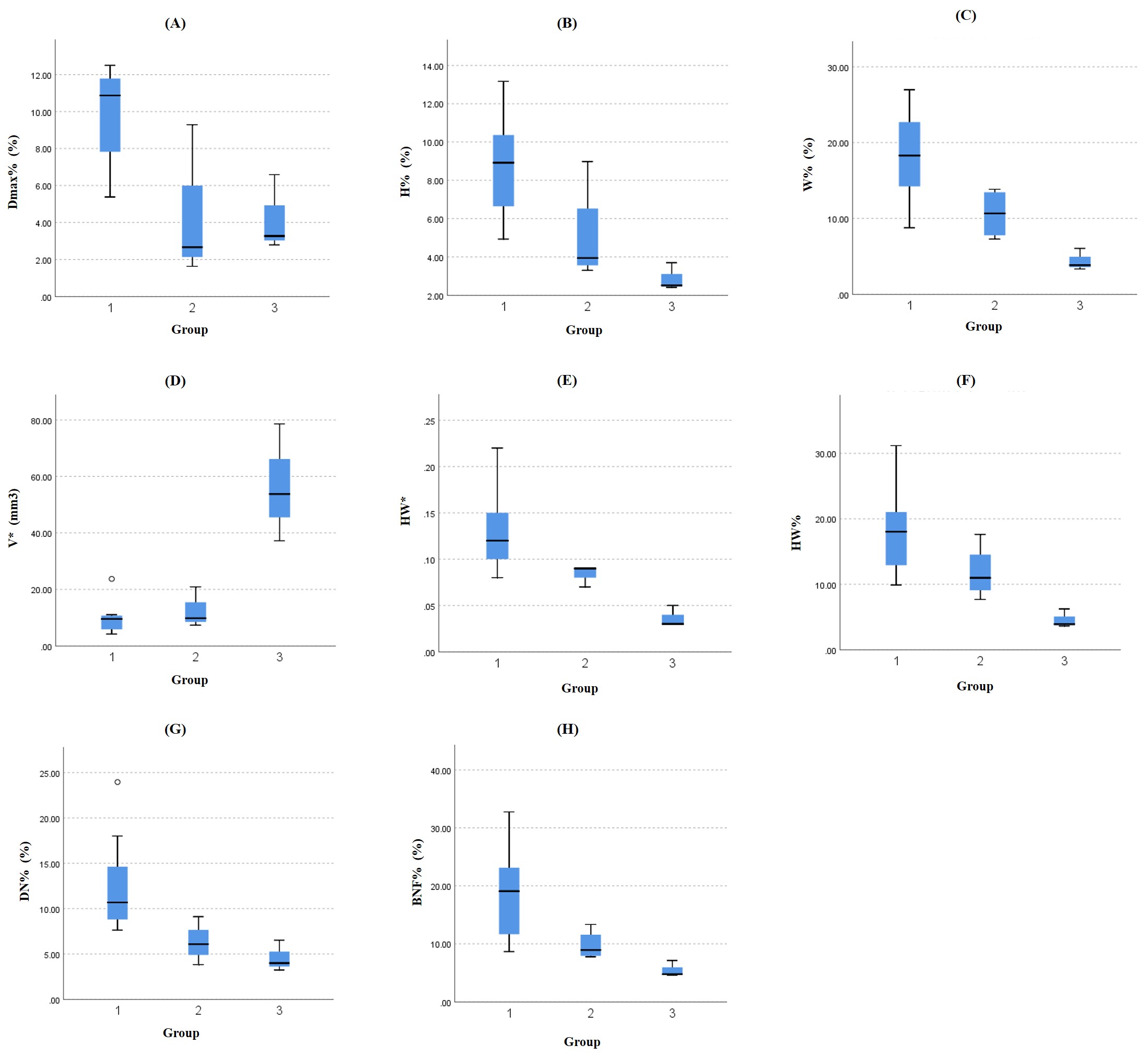


(A)

(B)

(C)

(D)

(E)

(F)

(G)

(H)

(I)


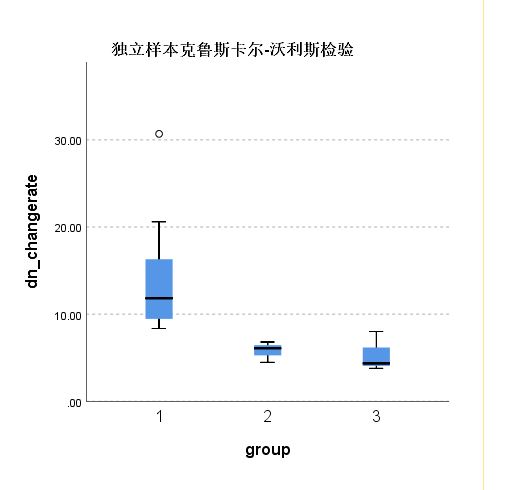

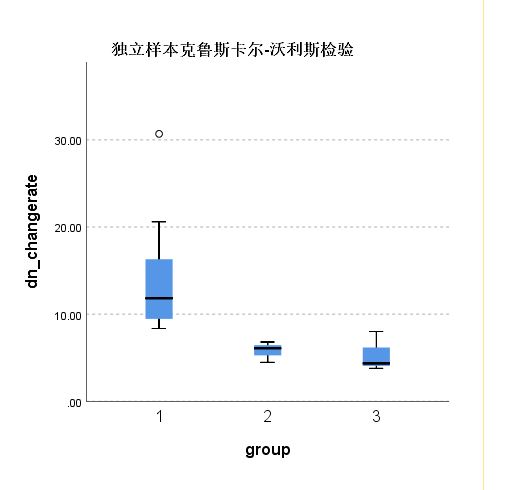

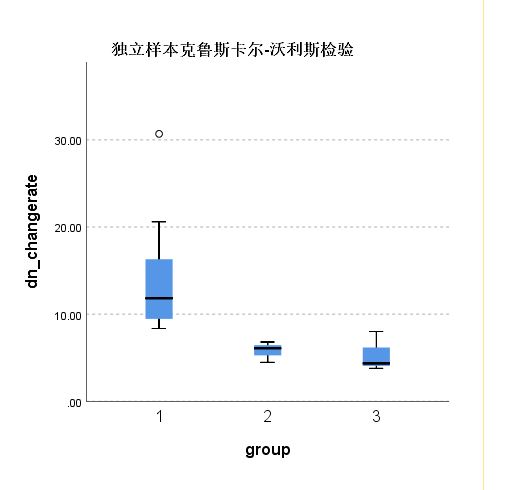


The differences in the relative change rate of (A) the maximum diameter, (B) aneurysm height, (C) aneurysm width, (D) aneurysm surface area, (G) height-to-width ratio, (H) dome-to-neck ratio, and (I) bottleneck factor, as well as the absolute change of (E) aneurysm volume, (F) height-to-width ratio among the three groups.

### Online Table 2 Differences in morphological parameters between aneurysms with irregular pulsation and global dilation

| Group | Irregular pulsation  (N = 7) | Global dilation  (N = 7) | P |
| --- | --- | --- | --- |
| Parameters |  |  |  |
| Location  MCA  ICA  PComA  VA | 2  0  1  4 | 3  3  1  0 | 0.073 |
| Dmax (mm) | 7.09$\pm$3.36 | 9.29$\pm$4.45 | 0.315 |
| Dmax* (mm) | 0.64$\pm$0.16 | 0.29$\pm$0.13 | **0.001** |
| Dmax% (%) | 10.22$\pm$2.12 | 3.62$\pm$1.86 | $\mathbf{<}$ **0.001** |
| H (mm) | 4.20 (3.33, 8.71) | 7.06 (2.61, 11.59) | 0.097 |
| H* (mm) | 0.33 (0.23, 0.56) | 0.27 (0.19, 0.30) | 0.073 |
| H% (%) | 8.38$\pm$3.08 | 4.45$\pm$2.65 | **0.025** |
| W (mm) | 6.45$\pm$2.42 | 8.36$\pm$4.13 | 0.311 |
| W* (mm) | 0.77$\pm$0.28 | 0.71$\pm$0.24 | 0.703 |
| W% (%) | 14.87$\pm$7.65 | 11.35$\pm$7.64 | 0.406 |
| S (mm^2^) | 71.97 (44.47, 356.25) | 154.11 (50.51, 458.94) | 0.209 |
| S* (mm^2^) | 7.87 (4.22, 21.46) | 5.97 (3.28, 17.21) | 0.318 |
| S% (%) | 10.04 (6.23, 22.46) | 3.96 (3.11, 7.36) | **0.002** |
| V (mm^3^) | 74.96 (33.96, 799.36) | 178.42 (44.56, 1022.90) | 0.165 |
| V* (mm^3^) | 10.00 (4.34, 78.58) | 11.06 (4.21, 53.77) | 0.902 |
| V% (%) | 13.72 (10.44, 30.67) | 6.36 (3.98, 11.49) | **0.002** |
| NW | 7.35$\pm$2.97 | 7.88$\pm$2.72 | 0.732 |
| NW* | 0.37 (0.25, 0.98) | 0.40 (0.29, 0.52) | 0.710 |
| NW% (%) | 4.68 (4.00, 17.87) | 6.21 (2.23, 8.87) | 0.902 |
| Dv (mm) | 2.80 (1.72, 3.52) | 2.86 (2.55, 4.80) | 0.620 |
| Dv* (mm) | 0.27 (0.18, 0.68) | 0.23 (0.11, 0.29) | 0.209 |
| Dv% (%) | 10.02 (5.42, 27.07) | 7.03 (2.44, 10.50) | 0.128 |
| HW | 0.76$\pm$0.17 | 0.89$\pm$0.13 | 0.138 |
| HW* | 0.11$\pm$0.06 | 0.08$\pm$0.03 | 0.345 |
| HW% (%) | 16.66$\pm$9.11 | 10.19$\pm$4.86 | 0.123 |
| DN | 0.52$\pm$0.47 | 0.94$\pm$0.50 | 0.132 |
| DN* | 0.05$\pm$0.04 | 0.05$\pm$0.03 | 0.883 |
| DN% (%) | 10.68 (6.52, 23.95) | 5.95 (3.23, 8.87) | **0.004** |
| BNF | 0.90$\pm$0.16 | 1.06$\pm$0.36 | 0.311 |
| BNF* | 0.08 (0.04, 0.25) | 0.09 (0.06, 0.14) | 0.620 |
| BNF% (%) | 11.92 (4.77, 32.76) | 8.66 (4.61, 24.08) | 0.318 |
| AR | 0.67$\pm$0.17 | 1.00$\pm$0.35 | **0.042** |
| AR* | 0.07$\pm$0.03 | 0.06$\pm$0.03 | 0.633 |
| AR% (%) | 10.67$\pm$4.40 | 6.22$\pm$3.12 | **0.050** |
| SR | 2.16$\pm$1.00 | 2.89$\pm$1.23 | 0.251 |
| SR* | 0.27 (0.14, 0.80) | 0.22 (0.08, 0.31) | 0.383 |
| SR% (%) | 13.72 (8.49, 24.58) | 7.13 (5.03, 14.25) | **0.007** |
| NSI | 0.08 (0.05, 0.14) | 0.09 (0.04, 0.23) | 0.805 |
| NSI* | 0.03 (0.01, 0.05) | 0.01 (0.01, 0.01) | **0.004** |
| NSI% (%) | 29.70 (21.88, 61.90) | 11.14 (5.39, 19.98) | **0.001** |
| Inflow Angle ($\boldsymbol{^{\circ})}$ | 104.63$\pm$51.53 | 125.73$\pm$41.98 | 0.417 |
| PHASES score | 4 (4, 10) | 6.29$\pm$2.22 | 0.423 |
| Juvela score | 2.43$\pm$2.44 | 4.86$\pm$1.86 | 0.058 |

**Note:** Continuous values conforming to a normal distribution are expressed as mean $\pm$ SD, whereas those that deviate from a normal distribution are represented by the median (range). The counting parameter is expressed as numbers. MCA, middle cerebral artery; ICA, internal carotid artery; PComA, posterior communicating artery; VA, vertebral artery. Dmax, aneurysm maximum diameter; H, aneurysm height; W, aneurysm width; S, aneurysm surface area; V, aneurysm volume; NW, neck width; Dv, the average diameter of parent arteries; HW, the height-to-width ratio; DN, the dome-to-neck ratio; BNF, the bottleneck factor; AR, aspect ratio; SR, size ratio; NSI, non-sphericity index. (parameter)* is the difference between the maximum and minimum values of a parameter in one cardiac cycle; (parameter)% is the ratio of the difference between the maximum value and the minimum value to the minimum value of a parameter in one cardiac cycle. The bold values indicate the statistically significant differences in the parameters among the three groups.
